# Understanding Consumption of Illicit Drugs and New Psychoactive Substances in Australia by Triangulating Drug Checking and Wastewater‐Based Epidemiology

**DOI:** 10.1002/dta.70120

**Published:** 2026-07-10

**Authors:** Muhammad Usman, Malcolm McLeod, Dhayaalini Nadarajan, Joseph Clokey, Pritesh Prasad, Rory Verhagen, Benjamin Tscharke, Maurizio Quinto, Richard Bade

**Affiliations:** ^1^ Queensland Alliance for Environmental Health Sciences The University of Queensland Dutton Park QLD Australia; ^2^ Department of Agriculture, Food, Natural Resources, and Engineering (DAFNE) University of Foggia Foggia Italy; ^3^ Research School of Chemistry Australian National University Acton ACT Australia; ^4^ The Australian Drug Observatory Australian National University Acton ACT Australia

**Keywords:** data triangulation, early warning system, pill testing, wastewater analysis

## Abstract

Consumption of psychotropic drugs is a major economic and public health challenge in Australia. Wastewater‐based epidemiology (WBE) and drug checking have emerged as complementary tools to monitor this market. WBE estimates drug use within communities for target compounds and their metabolites, while drug checking is a free service available in some Australian jurisdictions, which allows individuals to verify drug purity and composition. In this work, wastewater samples were collected from five sites across Queensland (*n* = 3), New South Wales (*n* = 1) and Victoria (*n* = 1) over the 2024–25 New Year holidays, while drug checking was carried out at a site in central Canberra over December–January. Wastewater samples were analysed for alcohol, illicit drugs and new psychoactive substances (NPS) using liquid chromatography–tandem mass spectrometry. Methamphetamine was the most consumed illicit substance across all sites, while cocaine and MDMA showed distinct increases over the New Year period. Among NPS, bromazolam and 2′‐fluoro‐2‐oxo‐PCE were most prevalent, while ketamine was consistently detected at regional sites with consumption 2.8–3‐fold higher on New Year's days. Drug checking revealed prevalence of MDMA, cocaine and ketamine. NPS were detected in 15%–20% of total drug samples submitted, with *N*,*N*‐dimethylpentylone and 2′‐fluoro‐2‐oxo‐PCE the most frequently found. This study is the first to demonstrate the potential synergy between WBE and drug checking, highlighting how their complementary roles can enhance drug surveillance, early warnings for emerging psychoactive substances and monitoring of changes in drug consumption during high‐risk periods.

## Graphical abstract

First Australian study combining wastewater‐based epidemiology and drug checking, demonstrating how complementary data sources enhance monitoring of illicit drugs and emerging psychoactive substances during high‐use periods.

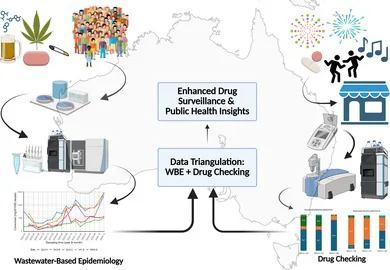

## 1 Introduction

There is considerable concern around the economic and social burden associated with the consumption of alcohol and illicit drugs in Australia. The National Drug Strategy Household Survey (NDSHS) 2022–23 reported that an estimated 10.2 million Australians aged 14 years and over (about 47% of the population) used an illicit drug at least once in their lifetime, including the nonmedical use of pharmaceuticals [1]. Preliminary data from the Australian Bureau of Statistics (ABS) indicated that there had been 1635 drug‐induced deaths in 2023, corresponding to a rate of 6.1 deaths per 100,000 population [1]. The Australian Criminal Intelligence Commission (ACIC) reported that Australians consumed 22.2 t of cocaine, heroin, MDMA and methamphetamine, with an estimated street value of approximately $11.5 billion, between August 2023 and August 2024 [2]. These statistics highlight that illicit drug consumption is a significant concern with widespread economic and public health implications. The rapid proliferation of new psychoactive substances (NPS) further complicates this scenario, as these novel drugs often lack comprehensive toxicological data, posing substantial risk due to their unknown pharmacological effects and potential for severe short‐ and long‐term impacts [3].

There are many complementary data sources that have been used to understand the unregulated drug market such as emergency department toxico‐surveillance programs [4, 5], syringe residue analysis [6, 7], cross‐sectional surveys [8], law enforcement data [9], illicit drug profiling data [10], drug checking [11, 12, 13] and wastewater analysis or wastewater‐based epidemiology (WBE) [14]. While each of these methods has inherent limitations, the triangulation of multiple data sources enables more comprehensive information to be obtained [15]. Wastewater analysis has shown complementary strength for understanding community drug consumption [16], with studies exploring its use together with surveys [17], data of seized illicit drugs [18], taxation data [19], driving under the influence of drugs and data from law enforcement officials [20], forensic toxicological data [21] and sales data of licit substances [22]. Previous Australian studies have demonstrated the value of triangulating multiple surveillance approaches to better understand illicit drug markets and emerging psychoactive substances [17, 18, 21].

Critical periods that see notable fluctuations in illicit drug consumption are major social events, public festivals, music events, the New Year period and sports events [14, 23, 24, 25, 26]. Studies have indicated that drug use often increases during festive periods due to heightened social gatherings, celebrations and changes in behavioural patterns [27]. One means to provide real‐time drug information at or around specific events of increased concern is through drug checking services. In Australia, on‐site drug checking has been provided at festivals in the Australian Capital Territory (ACT), New South Wales (NSW), Queensland and Victoria, while fixed‐site facilities have been operational since 2022 in the ACT, from 2024 to 2025 in Queensland, and in Victoria from mid‐2025. CanTEST, a fixed site drug checking service, tested 2939 samples and provided 4041 health, alcohol and other drug interventions during 2022–24 [28]. These services are provided freely to reduce harm by analysing substances submitted by the public and providing immediate results alongside personalised harm‐reduction advice [29]. In some drug checking implementations, the purity of samples can be determined, allowing clients to estimate dose and reduce the potential for overdose [28].

In this study, complementary insights were explored between WBE and drug checking data for monitoring illicit drugs and alcohol during the New Year holiday period in selected regional and capital city sites in Australia. Wastewater analysis was used to estimate population‐level consumption, while drug checking data provided information on the purity and identity of drugs used in episodic, event‐associated contexts. The results were compared with National Wastewater Drug Monitoring Program (NWDMP) and drug checking reports (CanTest and CheQpoint) to identify changes in drug consumption. The overall findings can provide insights into how both wastewater analysis and drug checking can be effectively used to monitor periods associated with increased drug consumption or changes to the substances circulating in the drug market during festive seasons that cannot be obtained from either method alone.

## 2 Methods

### 2.1 Wastewater Sampling

Wastewater sampling was conducted at five locations in three states of Australia. A total of 34 samples were collected from three urban and two regional locations from Queensland (Site‐A, Site‐B and Site‐C; *n* = 7 for each), NSW (Site‐D; *n* = 6) and Victoria (Site‐E; *n* = 7) for the 2024–2025 New Years period (the 7 days surrounding January 1st). Twenty‐four‐hour composite samples were collected for seven consecutive days from the inlet of wastewater treatment plants using automated sampling systems. Sampling dates and flow rates are shown in Table S1. Samples were acidified to pH 2 with 2 M hydrochloric acid on collection and refrigerated at 4°C during collection and kept on ice during shipment. The samples were stored at −20°C until analysis [30].

### 2.2 Chemicals of Interest

In the wastewater samples, the residues of ethyl sulphate (EtS) (metabolite of alcohol), methamphetamine, MDMA, MDA, benzoylecgonine (metabolite of cocaine) and 54 NPS [31] were targeted. The NPS were selected for monitoring based on information from Australian forensic laboratories as well as reports from the United Nations Office on Drugs and Crime and European Union Drugs Agency. A list of chemicals, drugs and internal standards (IS) used in this study is given in Table S2.

### 2.3 Chemical Analysis in Wastewater

The analytical methods applied in this study have been validated and published. For the analysis of alcohol and illicit drugs, filtered wastewater samples (1 mL) were spiked with isotopically labelled IS and analyzed without any extraction procedure [30]. All samples were analyzed by high‐performance liquid chromatography (Shimadzu Nexera HPLC system) coupled to a triple quadruple tandem mass spectrometer (SCIEX 5500 QTrap) for alcohol and (SCIEX 6500+ Triple Quad) LC–MS/MS for illicit drugs. Details of LC parameters are given in the supporting information. For the analysis of NPS, samples were extracted by solid phase extraction (SPE) using reported methods by Laimou‐Geraniou et al. [32]. Complete details of extraction procedure and LC parameters are given in the supporting information (Section S1), including the chromatographic separation of 2′‐fluoro‐2‐oxo‐PCE and 3′‐fluoro‐2‐oxo‐PCE (Table S3 and Figure S1). All analyses were performed using a SCIEX 7500+ Triple Quad (Toronto, Canada).

### 2.4 Back‐Calculation and Statistical Analysis

A back‐calculation was conducted to convert wastewater concentration measurements to consumption load estimates, where drug pharmacokinetic data were available (Equation 1) [33]. For MDA and NPS, this back‐calculation was not performed as reliable correction factors are currently unavailable. Instead, the mass loads for these drugs were calculated based on the concentration of the parent compounds (except ketamine, where the metabolite nor‐ketamine was analysed) and then population‐normalised to mg/day/1000 people (Equation 2) [34]. To avoid overestimation of MDA consumption, contributions from metabolism from MDMA were taken into account [35]. For alcohol, concentrations of EtS in wastewater were multiplied by the daily wastewater flow and resulting excreted amounts were then corrected using a correction factor. This value was then normalised by dividing by the catchment population (in thousands) and the density of ethanol at 25°C, yielding L consumed/day/1000 people (Equation 3) [36]. Daily flow rates were provided by wastewater treatment plant operators for each day of sampling, reported in Table S1 as population‐normalised flows (L/day/person) to maintain anonymity of participating sites. The correction factors and standard drug dose used to calculate the consumption of drugs is given in Table S4. Population data were from Census 2021, by matching specific catchment areas against the smallest census unit (mesh blocks) and accounting for population dispersion by normalising against the geographic national address file (GNAF), as previously suggested. To check the impact of New Year events on drug consumption, independent two‐sample *t*‐tests were performed comparing New Year days (31st December to 2nd January) to the non–New Year days (other days from the same sampling campaign). Calculations, statistical analysis and data visualisation were performed using Microsoft Excel 365 and RStudio 2025.05.0.

(1) $$\text{Drug consumpt}.\left[\frac{\mathrm{mg}/{\mathrm{day}}^{-1}}{1000\;\text{people}}\right]=\frac{\text{Concentration}\;\left[\mathrm{ng}\;L^{-1}\right]\;x\;\text{Flow Rate}\;\left[\mathrm{ML}\;{\mathrm{day}}^{-1}\right]}{\text{Population}}\;x\;\frac{\mathrm{MW}\;\text{ratio}}{\text{Excretion Factor}}$$

(2) $$\text{Drug excretion}\;\left[\frac{\mathrm{mg}/{\mathrm{day}}^{-1}}{1000\;\text{people}}\right]=\frac{\text{Concentration}\;\left[\mathrm{ng}\;L^{-1}\right]\;x\;\text{Flow Rate}\;\left[\mathrm{ML}\;{\mathrm{day}}^{-1}\right]}{\text{Population}}$$

(3) $$\text{Alcohol consumpt}.\left[\frac{L/{\mathrm{day}}^{-1}}{\text{people}}\right]=\frac{\frac{Co\text{ncentration}\;\left[\mathrm{ng}\;L^{-1}\right]\;x\;\text{Flow Rate}\;\left[\mathrm{ML}\;{\mathrm{day}}^{-1}\right]}{\text{Population}}\;x\;\frac{\mathrm{MW}\;\text{ratio}}{\text{Excretion Factor}}}{\text{Ethanol Density}\;\left[g\;{\mathrm{mL}}^{-1}\right]}$$

### 2.5 Drug Checking

This study also compares data obtained at the CanTEST Health and Drug Checking Service located in central Canberra over New Year period (1 Dec–31 Jan) associated with each year assessed in this study (2022, 2023 and 2024). Samples were collected and analysed on‐site by Fourier transform infrared (FTIR, Bruker Alpha II spectrometer) and ultra‐performance liquid chromatography‐photodiode array (UPLC‐PDA, Waters ACQUITY H‐class PLUS system) according to published methods [11]. Drug purity was determined for eight targeted substances, including MDMA, cocaine and ketamine, as previously described [37]. Briefly 1–2 mg of drug sample was weighed into a vial and dissolved in mobile phase eluant to 1 mg/mL concentration, diluted in mobile phase eluent to 0.1 mg/mL and filtered (0.2 um). Sample injections (1 μL) were resolved on gradient elution composed of 0.1% formic acid in water and methanol on an ACQUITY UPLC CSH C18 column (50 × 2.1 mm, 1.7 μm) held at 40°C, with detection at 285 nm (MDMA), 275 nm (cocaine) or 257 nm (ketamine). The purity of samples was calculated from the area under the curve by comparison with a six‐point calibration spanning the range 5%–100% free base drug. Independently prepared quality control samples at high (100%), medium (50%) and low (15%) concentrations were analysed before and after each batch of samples to assess method performance. Purity distributions obtained were typically non‐normal (Schapiro–Wilk test) and were analysed using a nonparametric median test conducted in SPSS (version 29.0.0.0 (241)). To provide additional contextual interpretation, data from CheQpoint drug checking service operating in Queensland between April 2024 to April 2025 and CanTEST annual report (July 2024 to July 2025) were also considered [38, 39]. As drug checking data were obtained from a fixed‐site service, the analysed samples may not be representative of the broader Australian drug supply or of substances consumed within the wastewater catchments included in this study.

## 3 Results and Discussion

### 3.1 Comparison of Wastewater Results From New Year Periods and Typical Periods

The most frequently detected substances were EtS, methamphetamine, MDMA and benzoylecgonine (100% detection frequency) followed by MDA (15%). As for the NPS, a range of substances were detected across sampling sites, including bromazolam, 2′‐fluoro‐2‐oxo‐PCE, methcathinone (all 100%), *N*,*N*‐dimethylpentylone (97%), nor‐ketamine (44%), pentylone (20%) and dipyanone (12%). The excreted mass loads of these substances with detection frequencies less than 100% varied between sampling locations. Nor‐ketamine was only found at Site‐B (28.6%), Site‐D (100%) and Site‐E (100%). Dipyanone was only found at Site‐D (66.7%) and pentylone at Site‐E (100%). Detailed detection frequencies and consumption estimates, or mass loads, are given in Table S5.

#### 3.1.1 Alcohol and Illicit Drugs

Alcohol, MDMA and cocaine all showed increasing use on New Year days (31 December–2 January) compared with other days (Figure 1), similar to previous work [40]. Methamphetamine, while having the highest mass loads, did not show such trends, with only Site‐D showing a difference, albeit with much higher levels after the New Year, likely associated with direct disposal rather than consumption. MDA was not detected at Site‐A; however, at all other detected sites, it appeared only on New Year's Eve 2024/25, with the highest consumption observed at Site‐B. The sporadic detection of MDA suggests occasional use or possibly the periodic substitution for expected MDMA in the illicit drug market. Higher MDMA (*t*‐test *p =* 0.039) and MDA (*t*‐test *p =* 0.042) levels were observed during the New Year period relative to non–New Year days, although these findings should be interpreted cautiously due to the limited sample size.

**FIGURE 1:**
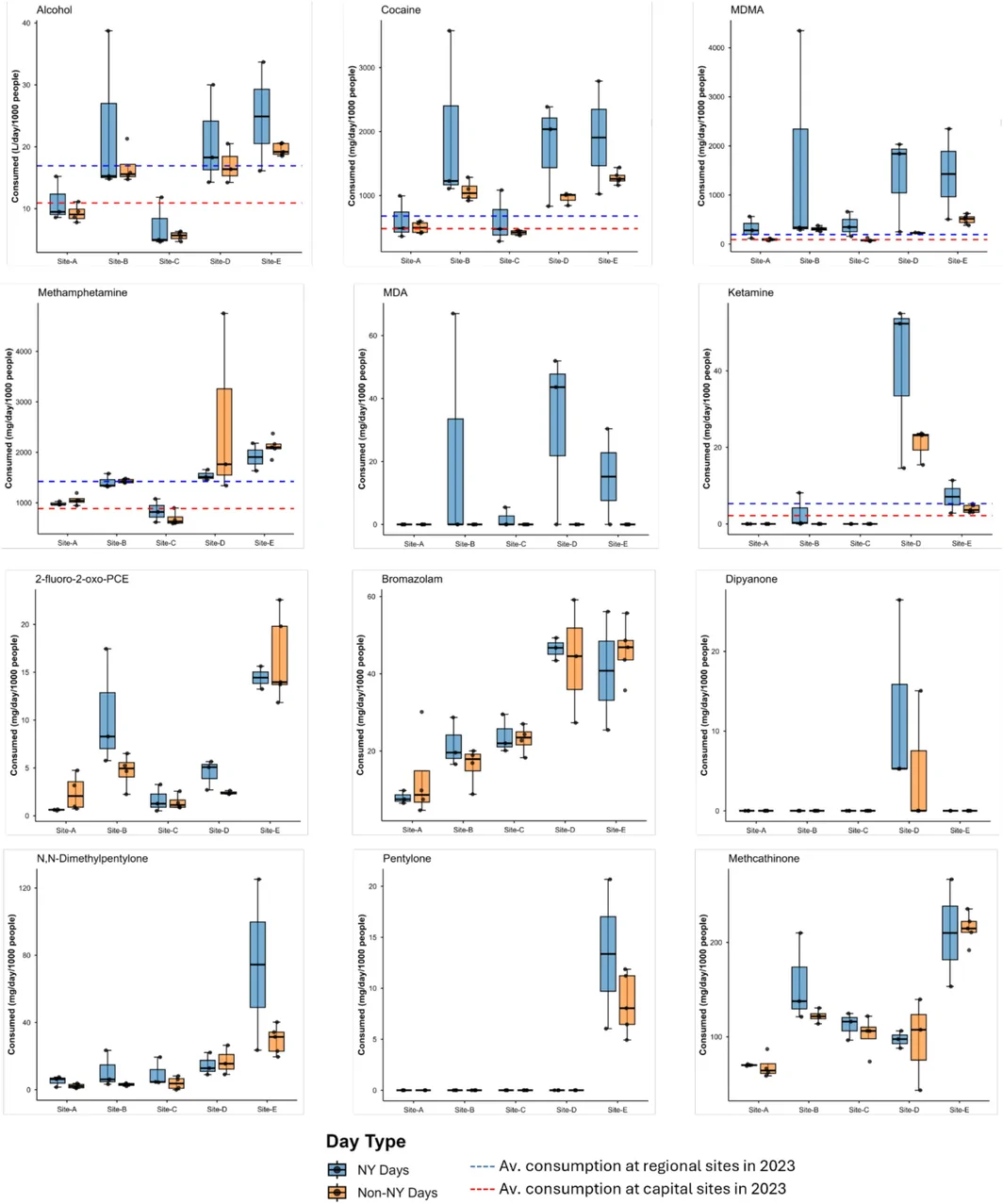
Comparison of alcohol, illicit drugs, and NPS consumed on New Year's days (December 31 to January 2) non–New Year's days (other sampling days) across five sites. Average consumption at regional (*n* = 2) and capital (*n* = 3) sites in 2023 is based on NDWMP data.

When compared with the NWDMP for December levels from the previous 2 years, the trends are quite similar, particularly for methamphetamine and alcohol (Figures S2 and S3), indicating that New Year events do not significantly alter their consumption from typical December weeks monitored for the NWDMP. However, Sites B, D and E all had substantial increases in MDMA compared with the NWDMP, potentially associated with music festivals or local events (Figure S4). Cocaine also showed an increase across all sites over the New Year compared with a typical December week, albeit not as great as MDMA (Figure S5).

When comparing the urban (Sites A, B and C) with the regional sites (Sites D and E), there were consistently higher levels of all illicit drugs and alcohol in the regional sites. While this was also reflected in the average NWDMP data, the difference across the New Year is far greater than during normal December weeks.

#### 3.1.2 NPS

Bromazolam and 2′‐fluoro‐2‐oxo‐PCE emerged as the most frequently detected NPS at all sites, whereas dipyanone and pentylone appeared more site specific. Bromazolam was found with the highest mass loads (average consumption ranged from 10.8 ± 8.7 to 45.1 ± 10.4 mg/day/1000 people). A 1.5‐fold increase in consumption of bromazolam was observed at Site‐B on New Year compared with other days (Figures 1 and S7). The level of excreted mass loads at regional sites was higher compared with capital city sites. Methcathinone has already been reported in Australia during New Year holidays 2019–20, but excreted mass loads are much higher during New Year period 2024–25 [41]. However, the detection of methcathinone in this study should be interpreted with caution. Previous studies in both Australia and China have demonstrated that methcathinone can be formed in‐sewer through the oxidation of ephedrine and pseudoephedrine rather than reflecting direct consumption [42, 43]. Therefore, the observed methcathinone signal could be attributed to in‐sewer transformation rather than direct use. Ketamine (measured as its metabolite nor‐ketamine) was not detected at the capital Site‐A and Site‐C. At capital Site‐B, it was observed only on 2 days during the New Year period (0.2 and 8.1 mg/day/1000 people). In contrast, ketamine consumption was persistent at the regional Site‐D and Site‐E. When compared with nonevent periods from the NWDMP, ketamine consumption appears to have decreased at the capital sites while increasing at the regional sites, particularly at Site‐D (Figure S6). The detection of the fluorinated ketamine analogue 2′‐fluoro‐2‐oxo‐PCE across all sites may point to emerging trends in arylcyclohexylamine use because it was first detected through drug checking in Canberra in 2022 [44, 45]. The highest consumption was observed at capital city Site‐B on New Year (2.6 times higher compared with other days), while at regional Site‐E, it was found at consistently high levels throughout the week. The average consumption of *N*,*N*‐dimethylpentylone ranged from 3.5 ± 2.6 mg/day/1000 people at Site‐A to 42.5 ± 37.2 mg/day/1000 people at Site‐E, and it was detected across all sites (detection frequencies are given in Table S4). When comparing the New Year versus non–New Year days, consumption levels were 4.6 folds and 4.3 folds higher on New Year days at Site‐B and Site‐E respectively (Figures 1 and S7). Overall, regional sites demonstrated greater diversity in detected NPS compared with capital/city sites, including unique substances like dipyanone and pentylone, only found at regional sites. These findings highlight the complex and evolving NPS landscape in nonmetropolitan regions.

### 3.2 Drug Checking Data

Drug checking provides a complementary approach to evaluate trends in the drug market through the analysis of samples submitted for analysis by members of the public. It can provide information on the identity and purity of drug samples circulating in the community, including the occurrence of adulteration or the substitution of expected substances. Well‐resourced drug checking services also provide early warning and monitoring of newly emerging substances that can be used to inform and target other monitoring systems such as toxico‐surveillance of emergency department presentations [11, 12, 46]. For the current study, data obtained at the CanTEST Health and Drug Checking Service located in central Canberra (ACT) were compared from New Year periods (1 Dec to 31 Jan) in 2022, 2023 and 2024. Although the ACT was not included in the wastewater analysis, it is a capital city, which can be considered comparable with the other capital city sites investigated through wastewater analysis the NWDMP [2], However, the reported results from the CheQpoint drugs checking service operating in Queensland were also included. The most frequently expected drugs presented over the three New Year periods included MDMA (30%–42% of expected drugs), cocaine (15%–19%), ketamine (11%–13%), followed by smaller proportions (< 7% each) of LSD, performance and image enhancing drugs (PIEDs), methamphetamine, benzodiazepines, heroin and a diverse range of other substances (12%–17%). This pattern is similar to that observed in full‐year data [39].

Findings from the CheQpoint drug checking service during 2024–2025 (Table S6) provide additional support. Although not specifically focusing on the New Year, similar trends were seen to the CanTEST data, including MDMA (33%), cocaine (17%) and ketamine (11%) from the 906 drug samples analysed. Approximately 10% of drug checking samples contained unexpected psychoactive substances, indicating the presence of adulteration or substitution within the drug supply [38]. A total of 14 NPS were detected in these Queensland drug checking samples, including bromazolam, dimethylpentylone and nitazenes (all NPS are presented in Table S6). Over the same time period, CanTEST tested 2072 samples during 21 July 2024 to 20 July 2025, identifying a wide range of substances, with MDMA (33.4%), cocaine (18.6%) and ketamine (14.2%) being the most common. The most frequently detected NPS included 2C‐B (22 detections), 2′‐fluoro‐2‐oxo‐PCE (10), 4‐MMC (9) and bromazolam (7) [39].

#### 3.2.1 Drug Checking Over 2024/25 New Year Period

Specifically interrogating the 2024/25 drug checking results, in total, 299 drug samples were submitted for analysis, with the primary expected drugs being MDMA (*n* = 91; 30%), cocaine (*n* = 54; 18%) and ketamine (*n* = 39; 13%). This was broadly consistent with patterns observed in wastewater data, with these three substances all showing increases over the New Year period. Public drug alerts or community notices were also issued, related to AB‐MDMBA in expected benzodiazepine samples, nitazenes in expected oxycodone pills, bromazolam and 2C‐B in expected cocaine and multiple high dose MDMA pills [47].

#### 3.2.2 Changes in Purity in Drug Checking Samples

The three most expected drugs, MDMA, cocaine and ketamine, provided evidence of changes in purity over the 3 years assessed (Figure 2). Samples containing MDMA showed similar median purities (68%–72%, *p* > 0.05) but a narrowing interquartile range. Median purity for cocaine significantly increased between 2022 and 2023 (23% to 76%, *Χ* ^2^ = 22.1, *p* < 0.001). This coincides with NWDMP data from 2022 to 2024 where there was a consistent increase observed in cocaine at the five sites (Figure S5). Ketamine showed a similar but less pronounced pattern of increased median purity from 2022 to 2023 (72% to 85%, *Χ* ^2^ = 6.53, *p* < 0.05). As noted above, this modest increase in median purity coincides with a decrease in ketamine consumption in capital cities but an increase in regional areas observed in 2022 and 2023 NWDMP data.

**FIGURE 2:**
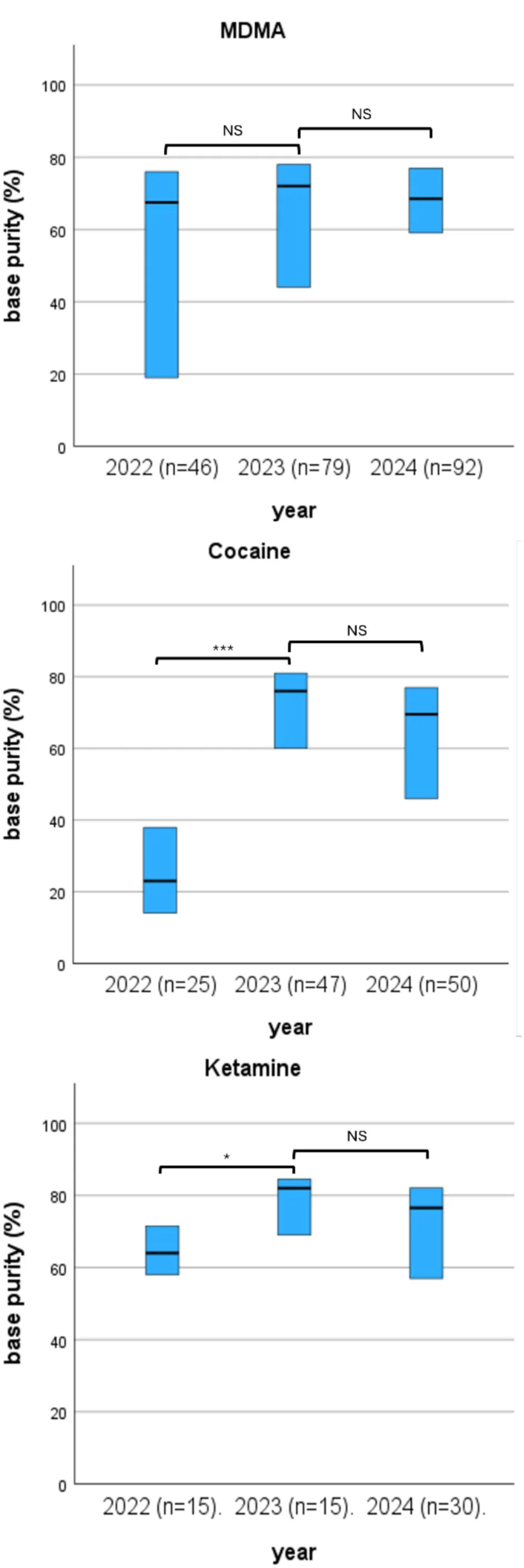
Median purity (Q2, % base) of major drugs presented to the CanTEST service over the New Year period (Dec–Jan) for each year of the study period. Boxes indicate the interquartile range (Q1–Q3). Statistical significance was assessed using a nonparametric median test; *p < 0.05; **p < 0.01; ***p < 0.001.

#### 3.2.3 Changes in Expected and Detected Drugs in Drug Checking Samples

Data for expected and detected drugs reveal patterns of drug adulteration or substitution that provide another window into the changing nature of drug samples over the same period. For each major expected drug, analytical findings were classified as containing (1) the expected drug only, (2) the expected drug in addition to some other psychoactive or non‐psychoactive substance, (3) some other psychoactive or non‐psychoactive substance or (4) no drug detected. Expected MDMA samples showed an increase in MDMA‐only detections and a reduction in substitution or cases of no drug detected, which co‐occurred with a decrease in the interquartile range observed above (Figure 3). Cocaine also showed a steady increase in cocaine‐only detections and a decrease in adulteration with other substances, which co‐occurred with a significant increase in median purity from 2022 to 2023. Ketamine showed an increase in ketamine‐only detections and a decrease in drug substitution in the last year (2024) surveyed relative to earlier years (2022, 2023). In this instance, ketamine median purity showed a modest increase between 2022 and 2023 and remained similar for 2024.

**FIGURE 3:**
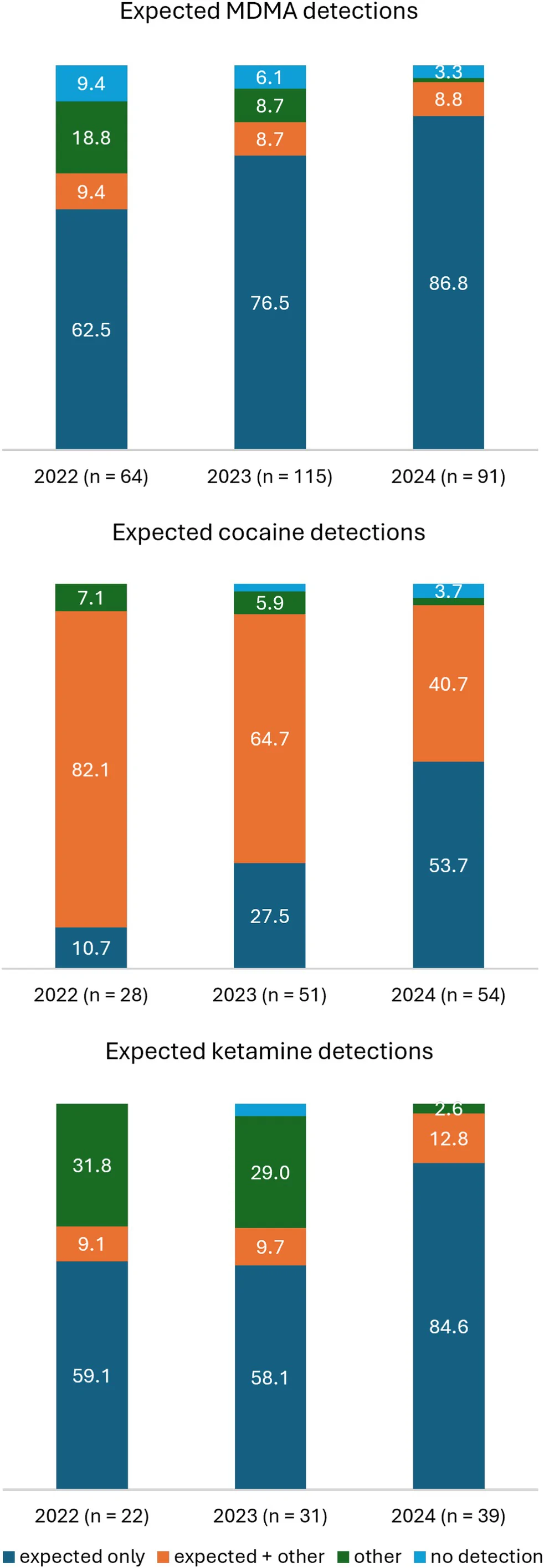
Analytical findings for major expected drugs MDMA, cocaine and ketamine. Data are presented as percentages and classified as expected drug only, expected drug plus other psychoactive or non‐psychoactive substance, other psychoactive or non‐psychoactive substance and no substance detected. The New Year period and number of samples (*n*) are given below.

#### 3.2.4 NPS in Drug Checking Samples

NPS made up a small but significant proportion of the drug detections in samples presented over the New year period (2022, 20%; 2023, 15%, 2024, 19%). These drugs can be found as adulterants in, or substituting for, commonly expected drugs, including MDMA, cocaine or ketamine, but also frequently presented and detected as expected drugs by members of the public. The most common NPS was ketamine that formed an increasing proportion of NPS detections over the study period (2022, 38%; 2023, 68%; 2024, 70%). This increase in NPS proportion occurred alongside a decrease in other NPS of the dissociative class, including 2′‐fluoro‐2‐oxo‐PCE (11%, 10%, 0%) and 2‐fluorodeschloroketamine (2‐FDCK, 8%, 0%, 2%). In total, drugs of the dissociative class made up the majority of NPS detected (68%, 83%, 74%) with smaller proportions of stimulants, psychedelics, sedative hypnotics and other NPS. The stimulant NPS *N*,*N*‐dimethylpentylone also showed a decrease in the proportion of NPS detected over the years surveyed (22%, 10%, 0%).

The detection of emerging NPS such as *N*,*N*‐dimethylpentylone, bromazolam and 2′‐fluoro‐2‐oxo‐PCE in the wastewater over this period underscores the evolving landscape of substance use at such events. They often substitute for other expected drugs such as MDMA, alprazolam (expected Xanax) and ketamine. For example, in 2024, a public health alert was issued in the ACT related to *N*,*N*‐dimethylpentylone, 2′‐fluoro‐2‐oxo‐PCE and MDMA in an expected MDMA pill [48]. In addition, pentylone and *N*,*N*‐dimethylpentylone have been reported at a music festival during 2022 in Australia, both with similar trends to MDMA, potentially indicating substitution or adulteration [17].

### 3.3 Complementary Insights From Drug Checking and Wastewater Data

The wastewater and drug checking datasets provide complementary insights. Increases in cocaine‐related wastewater loads occurred alongside higher cocaine purity (Figure 2) and reduced adulteration (Figure 3) observed in drug checking samples. However, the observed wastewater increases are more likely to reflect increased consumption during the New Year period. This interpretation is further supported by comparison with NWDMP data during routine monitoring periods, which showed consistently lower cocaine consumption than that observed during the New Year's campaign (Figure S5). In a similar manner, increased ketamine‐related wastewater loads at regional sites coincided with a greater proportion of ketamine samples identified as expected substances during drug checking (Figure 3), and a corresponding reduction in substitution and adulteration of ketamine with other NPS of the dissociative class (2′‐fluoro‐2‐oxo‐PCE and 2‐FDCK). These findings may reflect broader shifts in ketamine availability and market composition, although direct relationships between the datasets cannot be established. Increases in levels of MDMA detected in wastewater do not correspond to an increase in median purity for this substance at the drug checking service but coincided with reduced variability in drug checking purity distributions, suggesting greater consistency of MDMA available in the drug supply.

Despite methamphetamine representing the highest wastewater loads across all sites, it was infrequently encountered in drug checking submissions during the study period. This likely reflects differences in the populations, use contexts and behavioural patterns captured by each approach. Fixed‐site drug checking services are more commonly accessed by people using substances in episodic social or event‐associated settings, such as MDMA, cocaine and ketamine, whereas wastewater analysis captures broader population‐level consumption patterns, including substances associated with more regular or dependent patterns of use.

Several NPS, including 2C‐B, 2‐FDCK, 3‐CMC, 5‐MeO‐DMT, AB‐MDMSBA, bk‐2C‐B, clobromazolam, DMT, DMXE and methylclonazepam, were each detected at least once during drug checking services in 2024–25 (Table 1 and S6). However, none of these substances were detected in wastewater samples, potentially due to low consumption levels resulting in excreted mass loads below the limits of detection or quantification (LOD/LOQ). In contrast, 2′‐fluoro‐2‐oxo‐PCE and *N*,*N*‐dimethylpentylone showed decreased detections over the study period and were not detected through drug checking during the 2024 New Year period (Table 1) but were consistently observed in wastewater with detection frequencies of 100% and 97.1% across all sites, respectively. This contrast highlights the complementary nature of WBE and drug checking approaches. Drug checking detects substances directly submitted by users, and WBE provides population‐level insights, and together, they offer a more comprehensive understanding of overall drug consumption patterns. These substances, often with limited pharmacological data, pose significant public health challenges due to their unpredictable effects and potential for harm [49]. The consumption of both traditional illicit drugs and emerging NPS during holiday periods underscores the need to expand and strengthen existing harm reduction strategies to address evolving illicit drugs and NPS consumption. These may include expanding on‐site drug checking services at major events or festivals to identify the adulteration or substitution of drugs [50, 51], targeted education campaigns [52] and the availability of medical support to address potential overdoses or adverse reactions [53, 54]. Implementing such measures can help mitigate the health risks associated with drug use, healthcare burden and drug‐associated fatalities [55, 56]. The combined use of WBE and drug checking may therefore support earlier identification of emerging substances and improved situational awareness during periods associated with increased drug consumption.

**TABLE 1:** The NPS detected as a proportion of the total NPS detected in each New Year period. The New Year period and the total number of NPS detections are given as follows.

Compound	2022	2023	2024
2′‐Fluoro‐2‐oxo‐PCE	6 (10.8%)	4 (10.0%)	ND
4‐Bromo‐2,5‐dimethoxyphenethylamine (2C‐B)	2 (2.7%)	ND	1 (1.8%)
2‐Fluorodeschloroketamine (2‐FDCK)	5 (8.1%)	ND	1 (1.8%)
3‐Chloromethcathinone (3‐CMC)	2 (2.7%)	ND	1 (1.8%)
3‐Methoxyeticyclidine (3‐MeO‐PCE)	2 (2.7%)	ND	ND
5‐Methoxy‐*N*,*N*‐dimethyltryptamine (5‐MeO‐DMT)	ND	ND	1 (3.5%)
AB‐MDMSBA	ND	ND	1 (3.5%)
4‐Bromo‐2,5‐dimethoxy‐β‐ketophenylethylamine (bk‐2C‐B)	ND	ND	1 (1.8%)
Bromazolam	ND	1 (2.6%)	1 (1.8%)
Clobromazolam	ND	ND	1 (3.5%)
Dimethyltryptamine (DMT)	3 (5.4%)	ND	2 (5.3%)
Deoxymethoxetamine (DMXE)	ND	ND	1 (1.8%)
Ketamine	22 (37.8%)	27 (67.5%)	26 (70.2%)
Ketamine (+other)	2 (2.7%)	1 (2.5%)	ND
Mescalin	ND	1 (2.5%)	ND
Methylclonazepam	ND	ND	1 (1.8%)
*N*,*N*‐Dimethylpentylone	12 (21.6%)	4 (10.0%)	ND
*N*‐Ethyldeschloroketamine (2‐oxo‐PCE)	2 (2.7%)	1 (2.5%)	ND
Tiletamine	2 (2.7%)	ND	ND
Tramadol	ND	1 (2.5%)	1 (1.8%)
Total	57 (100.0%)	40 (100.0%)	37 (100.0%)

### 3.4 Limitations

This study has several limitations that should be acknowledged. The back‐calculation of drug consumption relies on assumptions about the contributing population. As the sampling period overlapped with the New Year holiday, the census‐based population estimates may not accurately reflect the true population within each catchment, as people may have temporarily moved to or away from these catchment areas. This introduces uncertainty into the calculated wastewater mass loads, which could result in over‐ or under‐estimation of actual consumption. Second, NWDMP data were used to compare event consumption with nonevent periods. This comparison was limited to illicit drugs only. For NPS, such monitoring data are not available, which prevented us from evaluating their long‐term temporal variation and distinguishing event‐related versus nonevent consumption. Drug checking data are presented for a single drug checking service located in central Canberra, ACT, that is geographically removed from the sites studied by wastewater. However, this drug checking data are the only data available that provide purity information and span the study period (2022–2024). Changes in drug purity and adulteration may reflect local changes in these measures that may not occur in locations where drug checking is not available or does not assess purity. The wastewater and drug checking datasets also represent different geographical areas, populations and behavioural contexts, limiting direct comparability between approaches.

Statistical comparisons between New Year and non‐New Year periods were exploratory and limited by the short sampling duration, small sample size and potential autocorrelation between consecutive daily wastewater samples.

## 4 Conclusion

This study combined WBE and drug checking data to understand the consumption patterns of alcohol, illicit drugs and NPS in Australia. Methamphetamine represented the highest wastewater loads across all sites but was rarely identified in drug checking submissions, while several NPS were only detected by drug checking, highlighting that WBE and drug checking capture different populations, use contexts and dimensions of illicit drug markets. The wastewater analysis also provided insights into regional areas, where drug checking in Australia currently fails to reach, with the two regional sites consistently showing higher mass loads of all drugs analysed.

Overall, the study demonstrates the value of WBE and drug checking as complementary tools for individual and population‐level monitoring of substance use. With most drug checking facilities in Australia based in capital cities, this study shows that regional areas also have increased use of illicit drugs and NPS during the festive season. Therefore, authorities may think to broaden drug checking to also include regional areas, especially at times of potentially higher drug use, such as the New Year and during music festival season. Moreover, this study highlights the knowledge gained from multiple data sources regarding the prevalence of illicit drugs and NPS in Australia, which can help to mitigate the health and societal harms associated with evolving drug consumption patterns in Australia.

## Author Contributions

Muhammad Usman: conceptualisation, writing – original draft, writing – review and editing, visualisation, data curation, formal analysis, investigation. Dhayaalini Nadarajan: methodology, investigation, writing – review and editing. Malcolm McLeod: conceptualisation, data curation, writing – original draft, writing – review and editing. Joseph Clokey: writing – review and editing. Pritesh Prasad: supervision, writing – review and editing. Rory Verhagen: supervision, writing – review and editing. Benjamin Tscharke: data curation, writing – review and editing. Maurizio Quinto: supervision, writing – review and editing. Richard Bade: conceptualisation, writing – review and editing, supervision, funding acquisition.

## Conflicts of Interest

MM is contracted by Directions Health Services to analyse drug samples derived from the CanTEST Health and Drug Checking Service.

## Supporting information


**TABLE S1:** Details of sampling campaign during 2024–25 New Year holidays.
**TABLE S2:** List of drug standards, internal standards and chemicals used.
**TABLE S3:** Mass spectrometric parameters for 2′‐fluoro‐2‐oxo‐PCE and 3′‐fluoro‐2‐oxo‐PCE.
**FIGURE S1:** Extracted ion chromatograms of 2′‐fluoro‐2‐oxo‐PCE and of 3′‐fluoro‐2‐oxo‐PCE. Top (A) chromatogram is a mixed standard (500 ng/L); lower (B) is showing a real wastewater sample containing of 2′‐fluoro‐2‐oxo‐PCE.
**TABLE S4:** Correction factors and standard drug dose used to calculate the consumptions.
**TABLE S5:** Substances consumed during New Year 2024–25 holidays (1average, 2standard deviation, 3minimum, 4maximum consumed values and 5detetction frequency). Where consumptions values (average, minimum and maximum) are in mg/day/1000 people for all illicit drugs and NPS and L/day/1000 people for alcohol.
**FIGURE S2:** Mean estimated consumption (L/day/1000 people) of alcohol at capital sites (A–C; shades of green) and regional sites (D and E; blue and gray) from 2022 to 2024. The blue dashed line represents the overall capital average, and the red solid line represents the overall regional average.
**FIGURE S3:** Mean estimated consumption (mg/day/1000 people) of methamphetamine at capital sites (A–C; shades of green) and regional sites (D and E; blue and gray) from 2022 to 2024. The blue dashed line represents the overall capital average, and the red solid line represents the overall regional average.
**FIGURE S4:** Mean estimated consumption (mg/day/1000 people) of MDMA at capital sites (A–C; shades of green) and regional sites (D and E; blue and gray) from 2022 to 2024. The blue dashed line represents the overall capital average, and the red solid line represents the overall regional average.
**FIGURE S5:** Mean estimated excretion (mg/day/1000 people) of cocaine at capital sites (A–C; shades of green) and regional sites (D and E; blue and gray) from 2022 to 2024. The blue dashed line represents the overall capital average, and the red solid line represents the overall regional average.
**FIGURE S6:** Mean estimated excretion (mg/day/1000 people) of ketamine at capital sites (A–C; shades of green) and regional sites (D and E; blue and gray) from 2022 to 2024. The blue dashed line represents the overall capital average, and the red solid line represents the overall regional average.
**FIGURE S7:** Trend of consumed drugs during sampling week across five sites by WBE. For consumption of alcohol, it is L/day/1000 people; for illicit drugs and NPS, it is mg/day/1000 people. Average consumption at regional (*n* = 2) and capital (*n* = 3) sites in 2023 is based on NDWMP data.
**TABLE S6:** Summary of drug checking results from the CheQpoint service (April 2024–April 2025). Data are presented as proportions of total psychoactive drug samples analyzed (*n* = 906). Data sourced from CheQpoint 12‐month evaluation report [5].
**SECTION S1:** Chemical analysis in wastewater.

## Data Availability

The data that support the findings of this study are available from the corresponding author upon reasonable request.
