# Supplementary material for: Understanding Consumption of Illicit Drugs and New Psychoactive Substances in Australia by Triangulating Drug Checking and Wastewater‐Based Epidemiology
Source: Drug Test Anal. 2026 Jul 10;18(10):1244–55. doi: 10.1002/dta.70120 (PMC13628436; doi:10.1002/dta.70120)
Supplement: Supplementary file 1 [file DTA-18-1244-s001.pdf]

**SUPPORTING INFORMATION**

# Understanding consumption of illicit drugs and new psychoactive substances in Australia by triangulating drug checking and wastewater-based epidemiology

Muhammad Usman[a,b], Malcolm McLeod[c,d], Dhayaalini Nadarajan[a], Joseph Clokey[a], Pritesh Prasad[a], Rory Verhagen[a], Benjamin Tscharke[a], Maurizio Quinto[b], Richard Bade[a]

[a] Queensland Alliance for Environmental Health Sciences, The University of Queensland, 20 Cornwall Street, Dutton Park, QLD 4102, Australia

[b] Department of Agriculture, Food, Natural resource, and Engineering (DAFNE), University of Foggia, via Napoli 25, I-71122 Foggia, Italy

[c] Research School of Chemistry, Australian National University, Acton, ACT 2601, Australia

[d] The Australian Drug Observatory, Australian National University, Acton, ACT 2601, Australia

***Table S1:*** *Details of sampling campaign during 2024-25 New Year holidays.*

| Site | Capital/Regional | Day | | Sampling Date | Population-normalized flows (L/day/person) |
|---|---|---|---|---|---|
| Site-**A** | ***Capital*** | ***Day-1*** | ***Saturday*** | **28-Dec-2024** | 223.53 |
| | | ***Day-2*** | ***Sunday*** | **29-Dec-2024** | 242.47 |
| | | ***Day-3*** | ***Monday*** | **30-Dec-2024** | 246.26 |
| | | ***Day-4*** | ***Tuesday*** | **31-Dec-2024** | 242.47 |
| | | ***Day-5*** | ***Wednesday*** | **01-Jan-2025** | 246.26 |
| | | ***Day-6*** | ***Thursday*** | **02-Jan-2025** | 238.68 |
| | | ***Day-7*** | ***Friday*** | **03-Jan-2025** | 242.47 |
| **Site-B** | *Capital* | *Day-1* | *Friday* | 27-Dec-2024 | 230.29 |
| | | *Day-2* | *Saturday* | 28-Dec-2024 | 248.79 |
| | | *Day-3* | *Sunday* | 29-Dec-2024 | 242.45 |
| | | *Day-4* | *Monday* | 30-Dec-2024 | 263.87 |
| | | *Day-5* | *Tuesday* | 31-Dec-2024 | 256.67 |
| | | *Day-6* | *Wednesday* | 01-Jan-2025 | 263.47 |
| | | *Day-7* | *Thursday* | 02-Jan-2025 | 239.32 |
| Site-**C** | ***Capital*** | **Day-1** | **Sunday** | **29-Dec-2024** | 225.26 |
| | | **Day-2** | **Monday** | **30-Dec-2024** | 214.85 |
| | | **Day-3** | **Tuesday** | **31-Dec-2024** | 231.60 |
| | | **Day-4** | **Wednesday** | **01-Jan-2025** | 227.50 |
| | | **Day-5** | **Thursday** | **02-Jan-2025** | 222.15 |
| | | **Day-6** | **Friday** | **03-Jan-2025** | 216.45 |
| | | **Day-7** | **Saturday** | **04-Jan-2025** | 208.90 |
| Site-**D** | *Regional* | Day-1 | *Friday* | 27-Dec-2024 | 304.51 |
| | | *Day-2* | *Saturday* | 28-Dec-2024 | 299.57 |
| | | *Day-3* | *Sample was not collected* | | |
| | | *Day-4* | *Monday* | 30-Dec-2024 | 291.17 |
| | | *Day-5* | *Tuesday* | 31-Dec-2024 | 310.03 |
| | | *Day-6* | *Wednesday* | 01-Jan-2025 | 311.48 |
| | | *Day-7* | *Thursday* | 02-Jan-2025 | 300.48 |
| Site-**E** | ***Regional*** | **Day 1** | **Thursday** | **26-Dec-2024** | 229.99 |
| | | ***Day-2*** | ***Friday*** | **27-Dec-2024** | 254.61 |
| | | ***Day-3*** | ***Saturday*** | **28-Dec-2024** | 276.64 |
| | | ***Day-4*** | ***Sunday*** | **29-Dec-2024** | 242.30 |
| | | ***Day-5*** | ***Monday*** | **30-Dec-2024** | 262.38 |
| | | ***Day-6*** | ***Tuesday*** | **31-Dec-2024** | 257.85 |
| | | ***Day-7*** | ***Wednesday*** | **01-Jan-2025** | 242.30 |

***Table S2:*** *List of drug standards, internal standards, and chemicals used.*

| Name | Category | Biomarker | CAS | Manufacturer |
|---|---|---|---|---|
| ***Ethyl Sulfate*** | Licit drug | Alcohol | 540-82-9 | Lipomed |
| ***Methamphetamine*** | Illicit drug | Methamphetamine | 7632-10-2 | Cerilliant |
| ***MDMA*** | Illicit drug | MDMA | 42542-10-9 | Cerilliant |
| ***MDA*** | Illicit drug | MDA | 4764-17-4 | Sigma Aldrich |
| ***Benzoylecognine*** | Illicit drug | Cocaine | 519-09-5 | Cayman Chemical |
| ***2'-fluoro-2-oxo-PCE*** | NPS | 2'-fluoro-2-oxo-PCE | 2850352-64-4 | Cayman Chemical |
| ***Bromazolam*** | NPS | Bromazolam | 71368-80-4 | Cayman Chemical |
| ***Dipyanone*** | NPS | Dipyanone | 60966-94-3 | Cayman Chemical |
| ***Nor-ketamine*** | NPS | Ketamine | 79499-59-5 | Cayman Chemical |
| ***Methcathinone*** | NPS | Methcathinone | 5650-44-2 | Cayman Chemical |
| ***N,N-Dimethylpentylone*** | NPS | *N,N*-Dimethylpentylone | 17763-13-2 | Cayman Chemical |
| ***Pentylone*** | NPS | Pentylone | 191471-79-9 | Cayman Chemical |
| ***Ethyl sulfate-D5*** | Internal standard | - | 118972-12-4 | Sigma Aldrich |
| ***Methamphetamine-D9*** | Internal standard | - | 169565-19-9 | Cerilliant |
| ***MDMA-D5*** | Internal standard | - | 1219795-77-5 | Cerilliant |
| ***MDA-D5*** | Internal standard | - | 1185282-84-1 | Cerilliant |
| ***Benzoylecgonine-D3*** | Internal standard | - | 115732-68-8 | Cerilliant |
| ***Nor-ketamine-D4*** | Internal standard | - | 1435934-57-8 | Cerilliant |
| ***Methanol*** | Solvent | - | 67-56-1 | Merck |
| ***Dichloromethane*** | Solvent | - | 75-09-2 | Merck |
| ***Isopropanol*** | Solvent | - | 67-63-0 | VWR Chemicals |
| ***Hydrochloric acid*** | Acid | - | 7647-01-0 | Merck |
| ***Formic acid*** | Acid | - | 64-18-6 | Fisher Chemical |
| ***Glacial acetic acid*** | Acid | - | 64-19-7 | VWR Chemicals |
| ***Sodium acetate*** | Base | - | 127-09-3 | VWR Chemicals |
| ***Ammonia*** | Base | - | 7664-41-7 | VWR Chemicals |

## Section S1: Chemical analysis in wastewater

### Ethanol metabolites in wastewater

Ethanol metabolite (EtS) was determined in wastewater using LC-MS/MS by adopting validated direst injection method [1]. Briefly, wastewater samples were acidified to pH = 2 at the time of collection, frozen and transported to the laboratory where they were stored frozen until preparation. The samples were defrosted for sample preparation and filtered using 0.2 µm cellulose syringe filters. The filtered sample (1 mL) was transferred into a 2 mL amber vial, and spiked with10 µL of 0.5 mg/L of internal standard (EtS-D5). After homogenization by vortex for 1 min, samples were analyzed using liquid chromatography (Shimadzu Nexera HPLC) tandem mass spectrometry (SCIEX, 5500-QTrap). The injection volume was 10 µL. The chromatographic separation was achieved using an Atlantis column (3 µm T3 column, 2.1 × 150 mm) at 40 °C with flow rate at 0.5 mL/min. 0.1 % formic acid in Milli-Q water (A) and 0.1 % formic acid in methanol (B) were used as mobile phase. Mobile phase linear gradient was set to: 0 % B at the first 2 min and increased to 95 % B in 1 min and stayed for 2 min, then dropped to 0 % B and stayed for 3 min for equilibration. The mass spectrometer operated in multiple reaction monitoring mode. Samples were reported as positive if the two transitions were present, retention time was within 0.15 minutes of the standard, and the relative intensity of the confirmation transition was within 20% of the expected value.

### Illicit drugs metabolites in wastewater

Illicit drugs metabolites were determined (as reported by Chen *et. al.* [2]) in wastewater by LC-MS/MS using a Shimadzu HPLC liquid chromatography system (Shimadzu Corp., Kyoto, Japan) coupled to SCIEX, 6500 + Triple Quad mass spectrometer (SCIEX, Concord, Ontario, Canada) equipped with an electrospray (TurboV) interface. The wastewater samples were defrosted and filtered using 0.2 µm cellulose syringe filters. The filtered sample (1 mL) was transferred into a 2 mL amber vial and spiked with 10 µL of 0.5 mg/L isotopically labeled internal standard mix. After homogenization by vortex, 7 microlitre injected into the column. Separation was achieved using a 2.6-micron 50x2.0 mm Phenomenex Kinetex Biphenyl column (Phenomenex, Torrance, CA) run at 45 ºC, and a flow rate of 0.45 mL $min^{-1}$ with a linear gradient starting at 5% B ramped to 100% B in 10.0 minutes then held at 100% for 4.5 minutes followed by equilibration at 5% B for 4.0 minutes. (A = 0.1% formic acid in HPLC grade water, B = 0.1% formic acid in methanol). A 50x4.6 mm, 5-micron Kinetex XB C18 column (Phenomenex, Torrance, CA) was inserted between the pumps and autosampler to trap mobile phase contaminants. The mass spectrometer was operated in scheduled multiple reaction-monitoring mode using nitrogen as the collision gas. Positive samples were confirmed by retention time and by comparing transition intensity ratios between the sample and an appropriate concentration standard from the same run. Samples were reported as positive if the two transitions were present, retention time was within 0.15 minutes of the standard, and the relative intensity of the confirmation transition was within 20% of the expected value. The value reported was that for the quantification transition.

### New psychoactive substances in wastewater

For the analysis of NPS, samples were extracted by solid phase extraction (SPE) using already reported method [3]. Briefly, 100 mL samples were filtered under vacuum using glass micro-fibre filter paper (GF/A 1.6 µm, Whatman, Kent, U.K.). Prior to solid phase extraction (SPE), the pH of the filtered samples was adjusted to 4.5–5 using aqueous ammonia (28 %) and surrogate internal standard (MDMA-d5, 150 ng/L) was then added to all samples. SPE cartridges (UCT XtracT DAU, 500 mg/6 mL; UCT Inc., Bristol, PA, USA) were conditioned with methanol (6 mL) and sodium acetate buffer (20 mM, 6 mL). The samples were then loaded under gravity, and the cartridges were successively washed with sodium acetate buffer (20 mM, 6 mL), 0.1 M acetic acid (2 mL) and methanol (6 mL). Analytes were eluted from the cartridges with a mixture of dichloromethane:isopropanol:aqueous ammonia (80:16:4 v/v/v, 6 mL) and evaporated to approximately 200 µL under nitrogen at 40 °C, and 1% HCl in methanol (20 µL) was added, then evaporated to dryness. The dry residue was reconstituted with 0.1% formic acid in methanol (20 µL) and 0.1% formic acid in ultrapure water (80 µL) to give a final concentration factor of 1000.

All analyses were performed using a Shimadzu Nexera LC (Kyoto, Japan) coupled to a SCIEX 7500 + Triple Quad MS (Toronto, Canada). A Kinetex biphenyl column (50 × 2.1 mm x 2.6 µm) was used for the chromatographic separation at a

flow rate of 0.45 mL/ min. The injection volume was 2 µL. The mobile phases used were 95% water with 5% methanol and 0.1% formic acid (solvent A) and 95% methanol with 5% water and 0.1% formic acid (solvent B). The initial percentage of B was 5%. After 1 min, it was increased linearly to 95% over 7 min and held for 2 mins. It was then reduced to 5% over 0.1 min where it was left for 1.9 min to equilibrate. The total run time was 11 min. The oven temperature was set to 40 °C. The MS system was operated in positive ionization in scheduled multiple reaction monitoring mode. The ion source parameters were as follows: 480 °C; curtain gas, 40 psi; collision gas 9 psi, medium; ion spray voltage, 2000 V; ion source gas 1 and ion source gas 2, 50 psi & 80 psi, respectively. Mass spectrometric analyses were performed in positive mode using multiple reaction monitoring (MRM). The two most abundant transitions of the precursor ion for each analyte and one for the deuterated internal standards were monitored.

To distinguish between 2′-fluoro-2-oxo-PCE and 3′-fluoro-2-oxo-PCE, we employed a Kinetex C8 column (150 x 2.1 mm x 2.6 µm), which allowed chromatographic separation of the two isomers using certified reference standards. The same chromatographic gradient as above was employed. Figure S1 shows the separation. The mass spectrometric parameters are shown below:

***Table S3:*** *Mass spectrometric parameters for 2'-fluoro-2-oxo-PCE and 3'-fluoro-2-oxo-PCE.*

| Compound | Retention Time | Q1 | Q3 | EP | CE | CXP | Q0D |
|---|---|---|---|---|---|---|---|
| 2′-fluoro-2-oxo-PCE | 4.43 | 235.8 | 83.0; 191.0 | 10 | 77; 19 | 12; 12 | -10; -10 |
| 3′-fluoro-2-oxo-PCE | 4.58 | 235.8 | 109.0; 83.0 | 10 | 48; 79 | 18; 25 | -10; -10 |

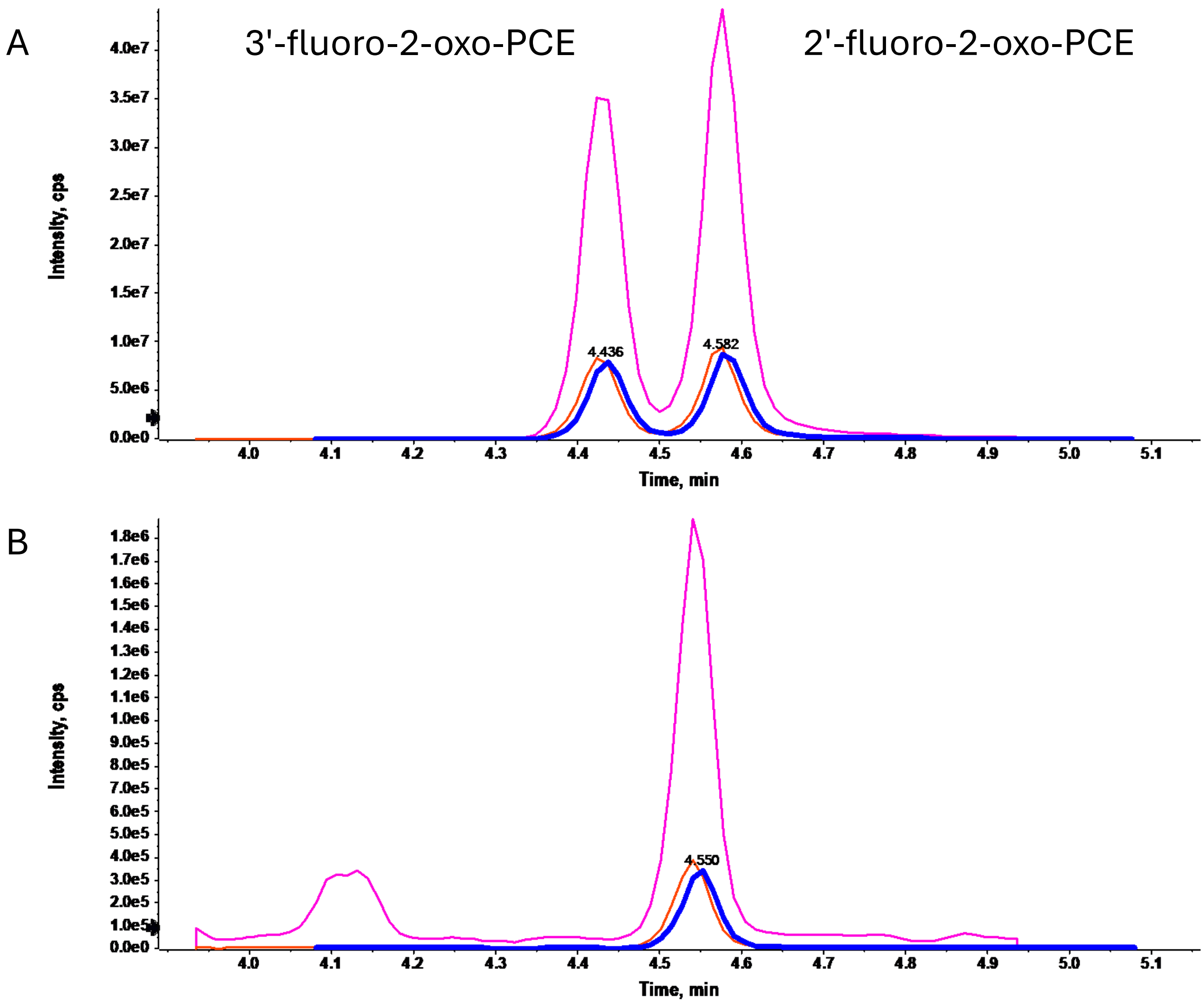


***Figure S1**: Extracted Ion Chromatograms of 2'-fluoro-2-oxo-PCE and of 3'-fluoro-2-oxo-PCE. Top (A) chromatogram is a mixed standard (500 ng/L), lower (B) is showing a real wastewater sample containing of 2'-fluoro-2-oxo-PCE.*

***Table S4:*** *Correction factors and standard drug dose used to calculate the consumptions.*

| Analyte | Drug | Excretion factor | STD dose pure drug (mg) | Ref |
|---|---|---|---|---|
| ***MDMA*** | MDMA | 0.225 | 100 | [4] |
| ***MDA*** | MDA | n.a. | n.a. | [4] |
| ***Methamphetamine*** | Methamphetamine | 0.39 | 30 | [4] |
| ***Ethyl sulfate*** | Alcohol | 0.00012 | 10 | [4] |
| ***Benzoylecgonine*** | Cocaine | 0.35 | 100 | [4] |
| ***Norketamine*** | Ketamine | n.a. | n.a. | [4] |

***Table S5:*** *Substances consumed during New Year 2024-25 holidays ([1]average, [2]standard deviation, [3]minimum, [4]maximum consumed values, and [5]detetction frequency). Where consumptions values (average, minimum, and maximum) are in mg/day/1000 people for all illicit drugs & NPS, and L/day/1000 people for alcohol.*

| *Site* | *Substance* | *Category* | *Consumption[1]* | *SD[2]* | *Min[3]* | *Max[4]* | *DF %[5]* |
|---|---|---|---|---|---|---|---|
| *A* | Cocaine (measured as benzoylecgonine) | Illicit | 5.5 | 2.1 | 3.6 | 9.9 | 100 |
| *B* | | | 14.6 | 9.4 | 9.2 | 35.8 | 100 |
| *C* | | | 5 | 2.6 | 2.9 | 10.8 | 100 |
| *D* | | | 13.6 | 6.8 | 8.3 | 23.9 | 100 |
| *E* | | | 14.6 | 6 | 10.2 | 27.9 | 100 |
| *A* | MDMA | Illicit | 1.9 | 1.8 | 0.7 | 5.6 | 100 |
| *B* | | | 8.9 | 15.2 | 2.6 | 43.5 | 100 |
| *C* | | | 2.1 | 2.2 | 0.6 | 6.6 | 100 |
| *D* | | | 8 | 8.8 | 2.2 | 20.3 | 100 |
| *E* | | | 7.7 | 7. | 3.8 | 23.5 | 100 |
| *A* | MDA | Illicit | N.D. | | | | |
| *B* | | | 9.6 | 23.4 | 0 | 67 | 14.3 |
| *C* | | | 0.8 | 1.9 | 0 | 5.5 | 14.3 |
| *D* | | | 15.9 | 22.6 | 0 | 51.9 | 33.3 |
| *E* | | | 4.3 | 10.6 | 0 | 30.4 | 14.3 |
| *A* | Methamphetamine | Illicit | 34.2 | 2.8 | 31.5 | 39.9 | 100 |
| *B* | | | 47.5 | 2.9 | 44 | 52.6 | 100 |
| *C* | | | 25.1 | 6.2 | 19.8 | 35.9 | 100 |
| *D* | | | 69.3 | 43.9 | 44.7 | 158.4 | 100 |
| *E* | | | 68.5 | 8 | 54.5 | 79.1 | 100 |
| *A* | Alcohol (measured as ethyl sulfate) | Licit | 10 | 2.3 | 7.8 | 15.2 | 100 |
| *B* | | | 19.4 | 8.1 | 14.7 | 38.7 | 100 |
| *C* | | | 6.3 | 2.3 | 4.6 | 11.8 | 100 |
| *D* | | | 18.9 | 5.4 | 14.2 | 30 | 100 |
| *E* | | | 21 | 5.3 | 16.1 | 33.7 | 100 |
| *A* | 2’-fluoro-2-oxo-PCE | NPS | 1.6 | 1.7 | 0.5 | 4.7 | 100 |
| *B* | | | 7.2 | 4.9 | 2.2 | 17.4 | 100 |
| *C* | | | 1.5 | 1 | 0.5 | 3.3 | 100 |
| *D* | | | 3.4 | 1.5 | 2.3 | 5.7 | 100 |
| *E* | | | 15.8 | 3.9 | 11.8 | 22.6 | 100 |
| *A* | Bromazolam | NPS | 10.8 | 8.7 | 4.7 | 30.1 | 100 |
| *B* | | | 18.5 | 5.9 | 8.8 | 28.7 | 100 |
| *C* | | | 23.4 | 3.9 | 18.3 | 29.5 | 100 |
| *D* | | | 45.1 | 10.4 | 27.3 | 59.1 | 100 |
| *E* | | | 44.6 | 11 | 25.4 | 56.1 | 100 |
| *A* | Dipyanone | NPS | N.D. | | | | |
| *B* | | | N.D. | | | | |
| *C* | | | N.D. | | | | |
| *D* | | | 8.7 | 10.3 | 0 | 26.5 | 66.7 |
| *E* | | | N.D. | | | | |
| *A* | Ketamine (measured as norketamine) | NPS | N.D. | | | | |
| *B* | | | 1.2 | 2.8 | 0.3 | 8.1 | 28.6 |
| *C* | | | N.D. | | | | |
| *D* | | | 30.7 | 18.2 | 14.6 | 55 | 100 |
| *E* | | | 4.8 | 3 | 2.8 | 11.4 | 100 |

| | | | | | | | |
|---|---|---|---|---|---|---|---|
| *A* | Methcathinone | NPS | 69.1 | 9 | 58.7 | 87 | 100 |
| *B* | | | 136.7 | 33.3 | 113.8 | 210 | 100 |
| *C* | | | 106.5 | 17.5 | 73.8 | 124.7 | 100 |
| *D* | | | 97 | 31.5 | 43.3 | 139.6 | 100 |
| *E* | | | 213.6 | 35.4 | 153.3 | 266.9 | 100 |
| *A* | *N,N*-Dimethylpentylone | NPS | 3.5 | 2.6 | 0.8 | 7.5 | 100 |
| *B* | | | 6.5 | 7.6 | 2.1 | 23.4 | 100 |
| *C* | | | 6.3 | 6.4 | 0 | 19.4 | 85.7 |
| *D* | | | 15.8 | 7.1 | 9 | 26.4 | 100 |
| *E* | | | 42.5 | 37.2 | 19.6 | 125.2 | 100 |
| *A* | Pentylone | NPS | N.D. | | | | |
| *B* | | | N.D. | | | | |
| *C* | | | N.D. | | | | |
| *D* | | | N.D. | | | | |
| *E* | | | 9.9 | 5.4 | 4.9 | 20.7 | 100 |

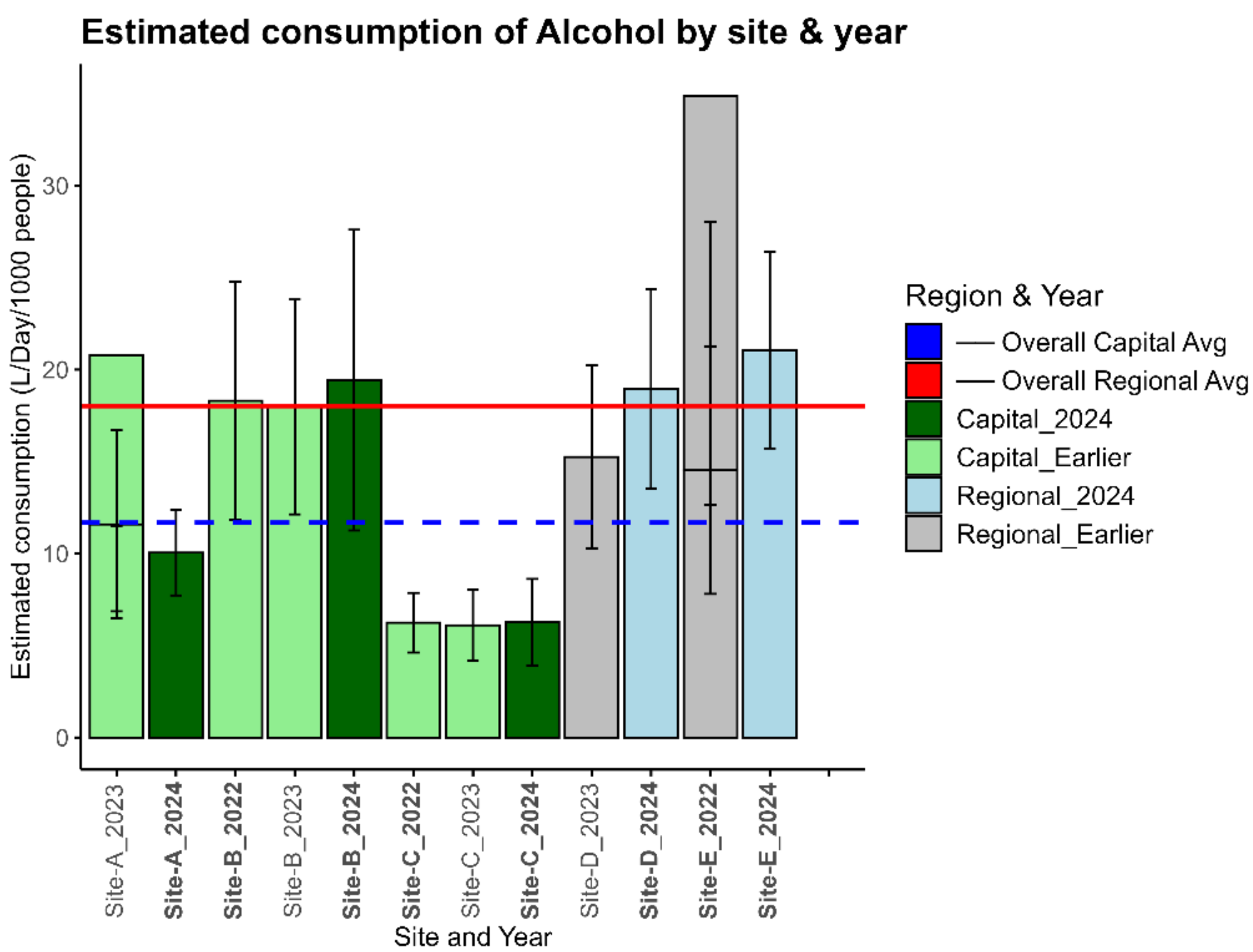


***Figure S2:*** *Mean estimated consumption (L/day/1000 people) of alcohol at capital sites (A–C; shades of green) and regional sites (D and E; blue and gray) from 2022 to 2024. The blue dashed line represents the overall capital average, and the red solid line represents the overall regional average.*

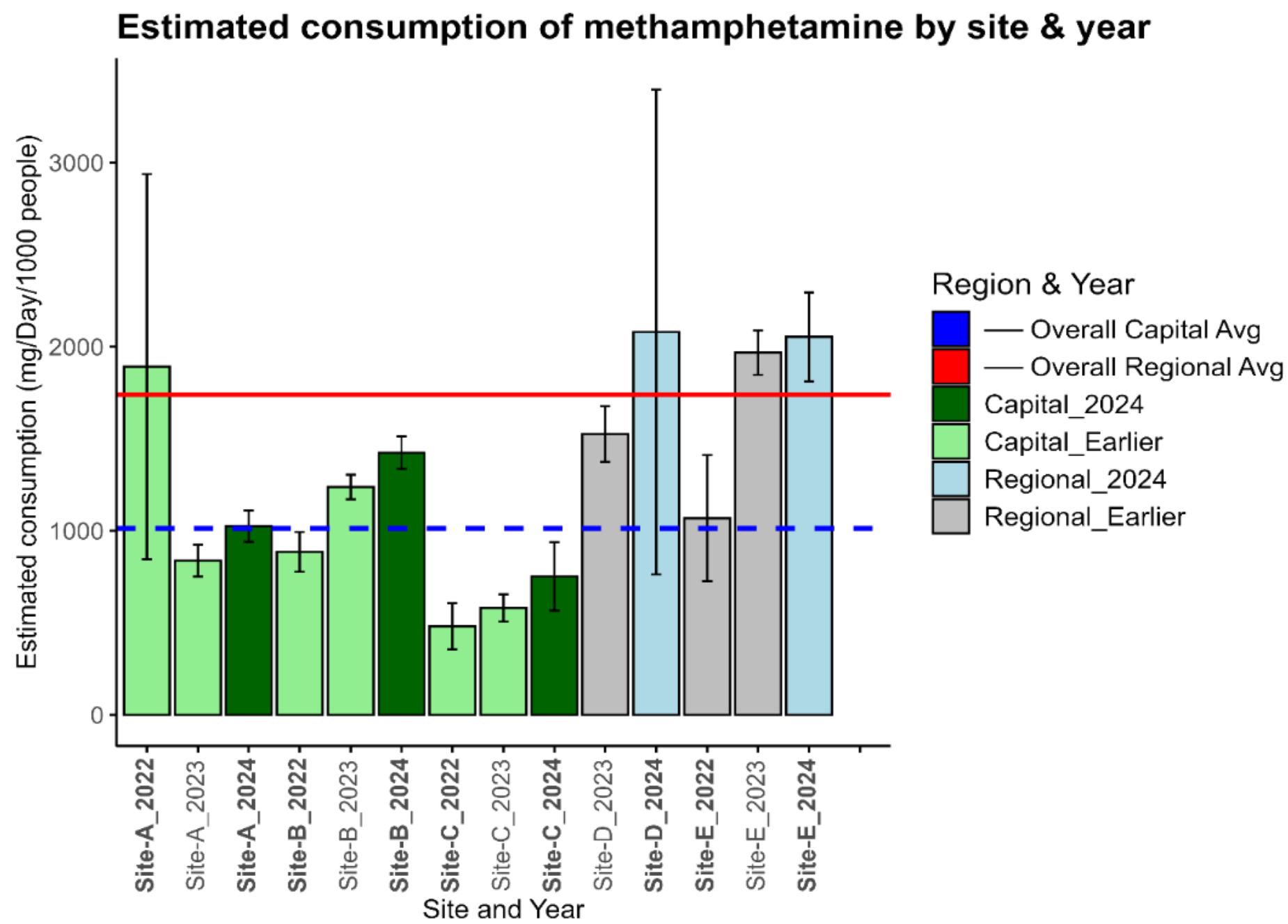


***Figure S3:*** *Mean estimated consumption (mg/day/1000 people) of methamphetamine at capital sites (A–C; shades of green) and regional sites (D and E; blue and gray) from 2022 to 2024. The blue dashed line represents the overall capital average, and the red solid line represents the overall regional average.*

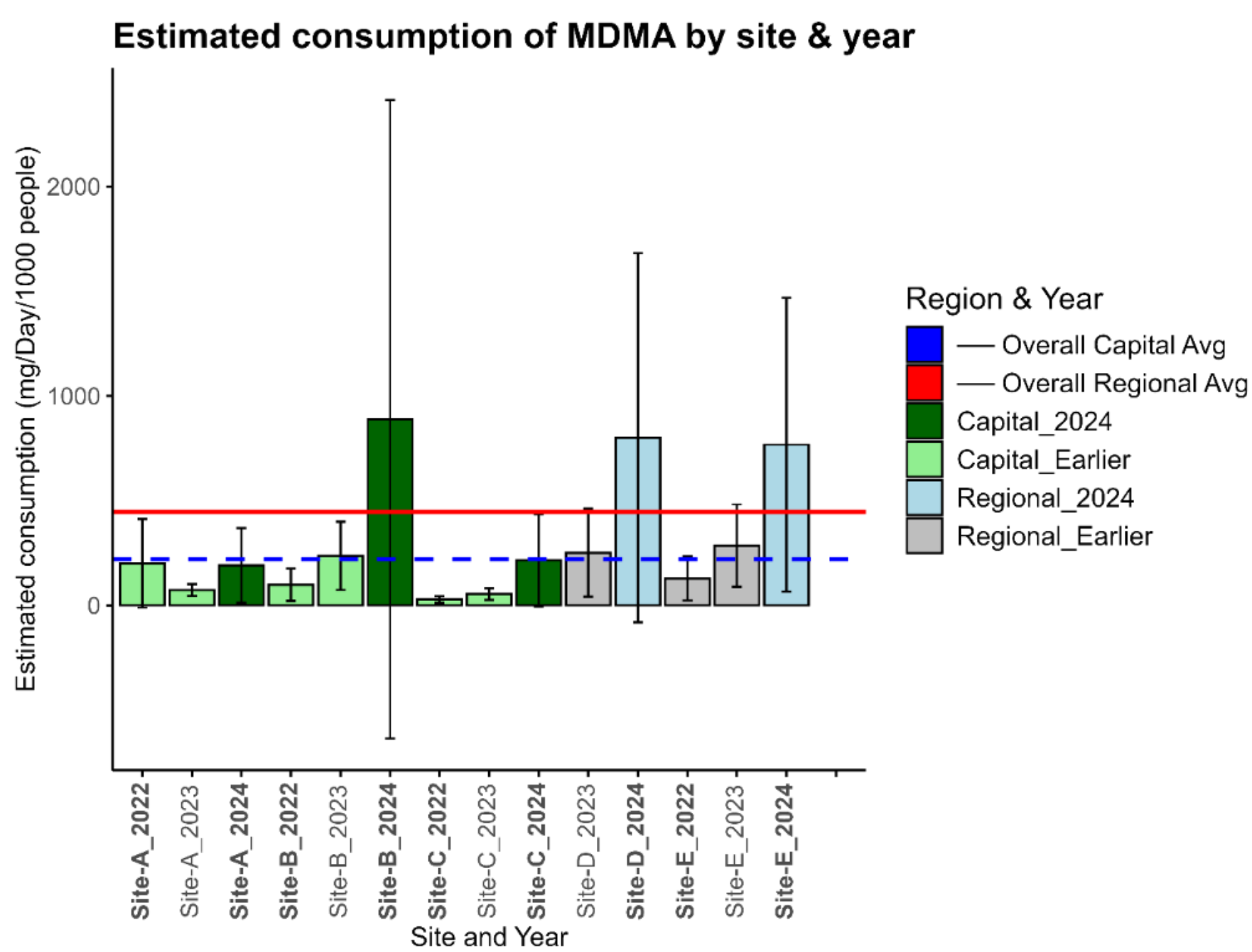


***Figure S4:*** *Mean estimated consumption (mg/day/1000 people) of MDMA at capital sites (A–C; shades of green) and regional sites (D and E; blue and gray) from 2022 to 2024. The blue dashed line represents the overall capital average, and the red solid line represents the overall regional average.*

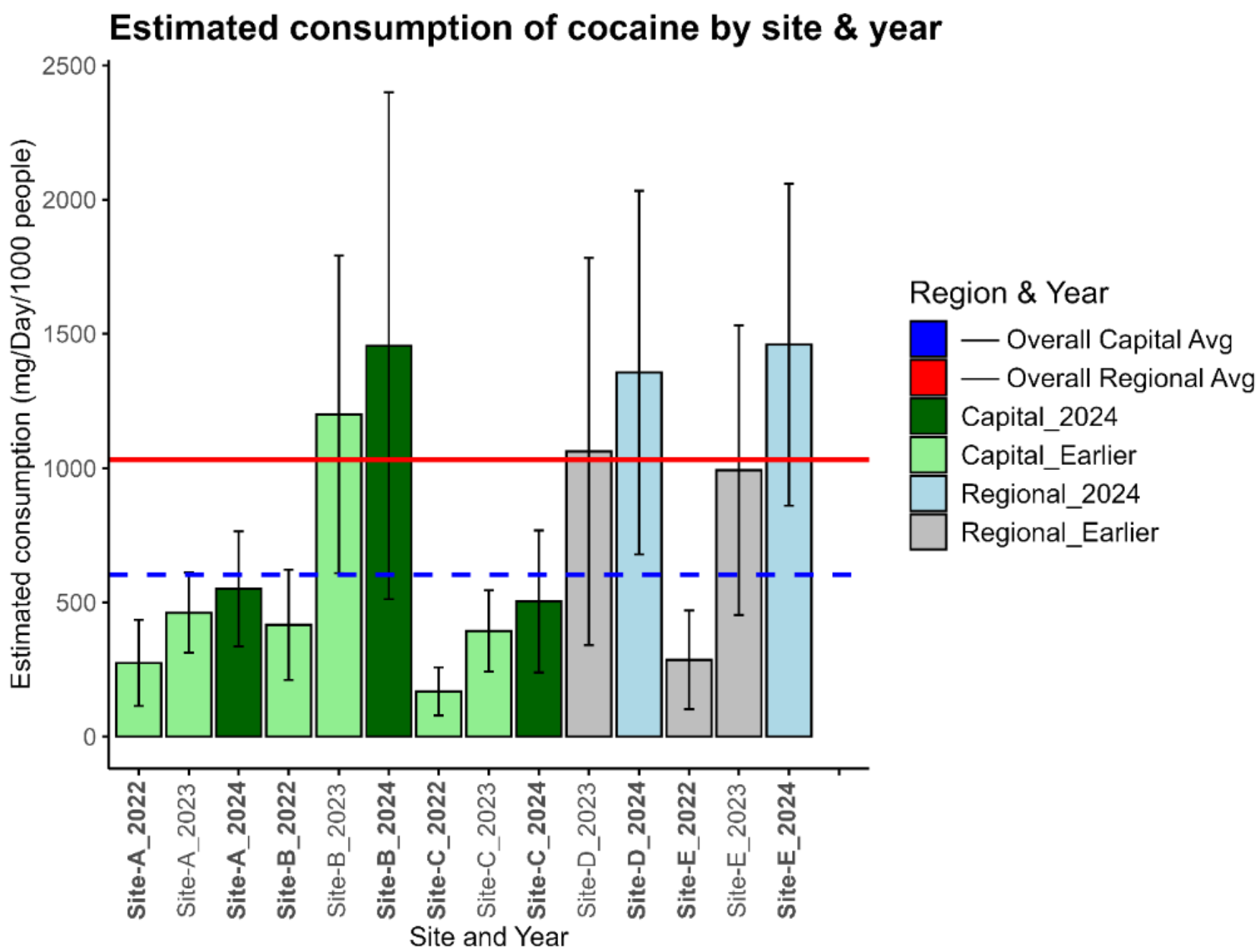


***Figure S5:*** *Mean estimated excretion (mg/day/1000 people) of cocaine at capital sites (A–C; shades of green) and regional sites (D and E; blue and gray) from 2022 to 2024. The blue dashed line represents the overall capital average, and the red solid line represents the overall regional average.*

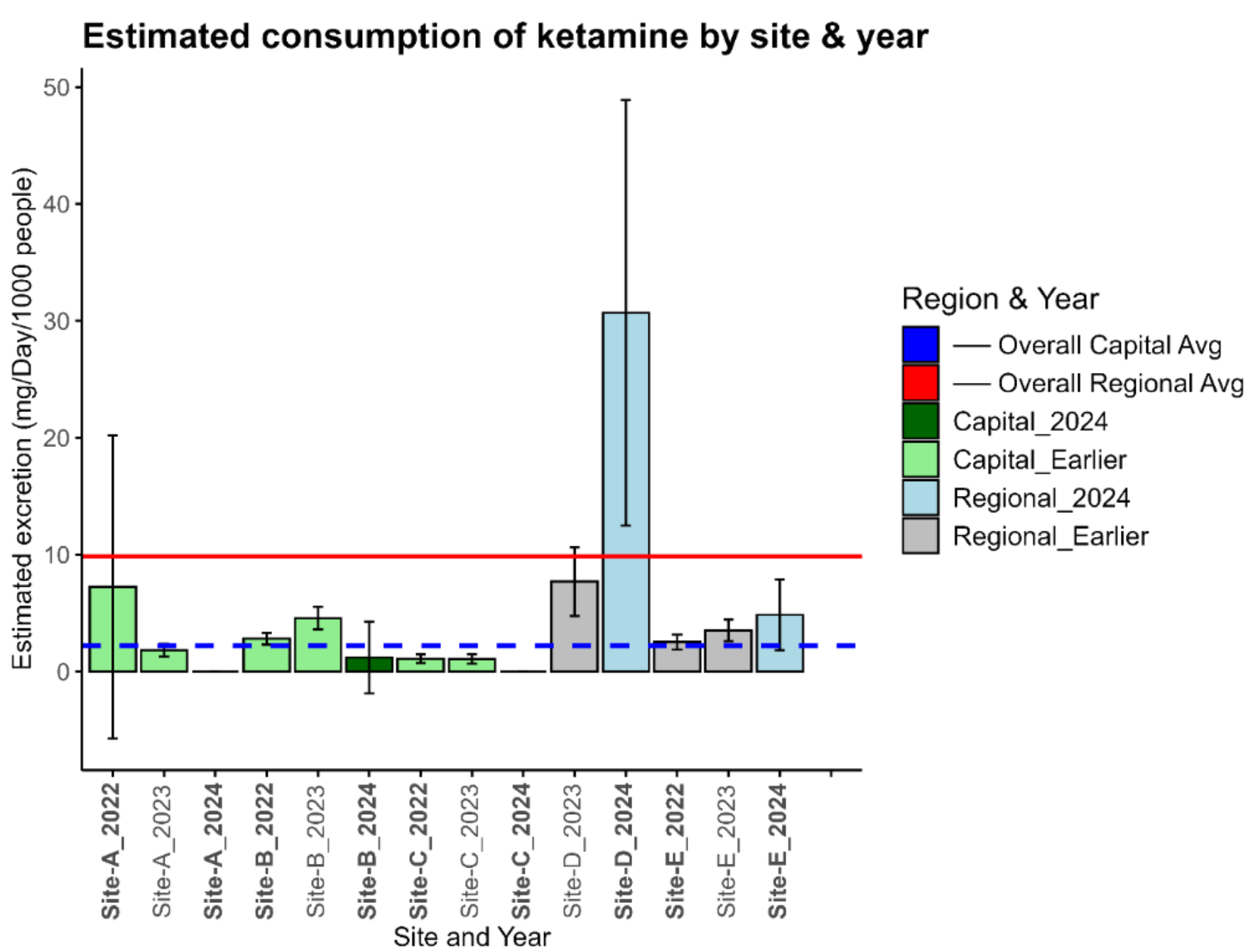


***Figure S6:*** *Mean estimated excretion (mg/day/1000 people) of ketamine at capital sites (A–C; shades of green) and regional sites (D and E; blue and gray) from 2022 to 2024. The blue dashed line represents the overall capital average, and the red solid line represents the overall regional average.*

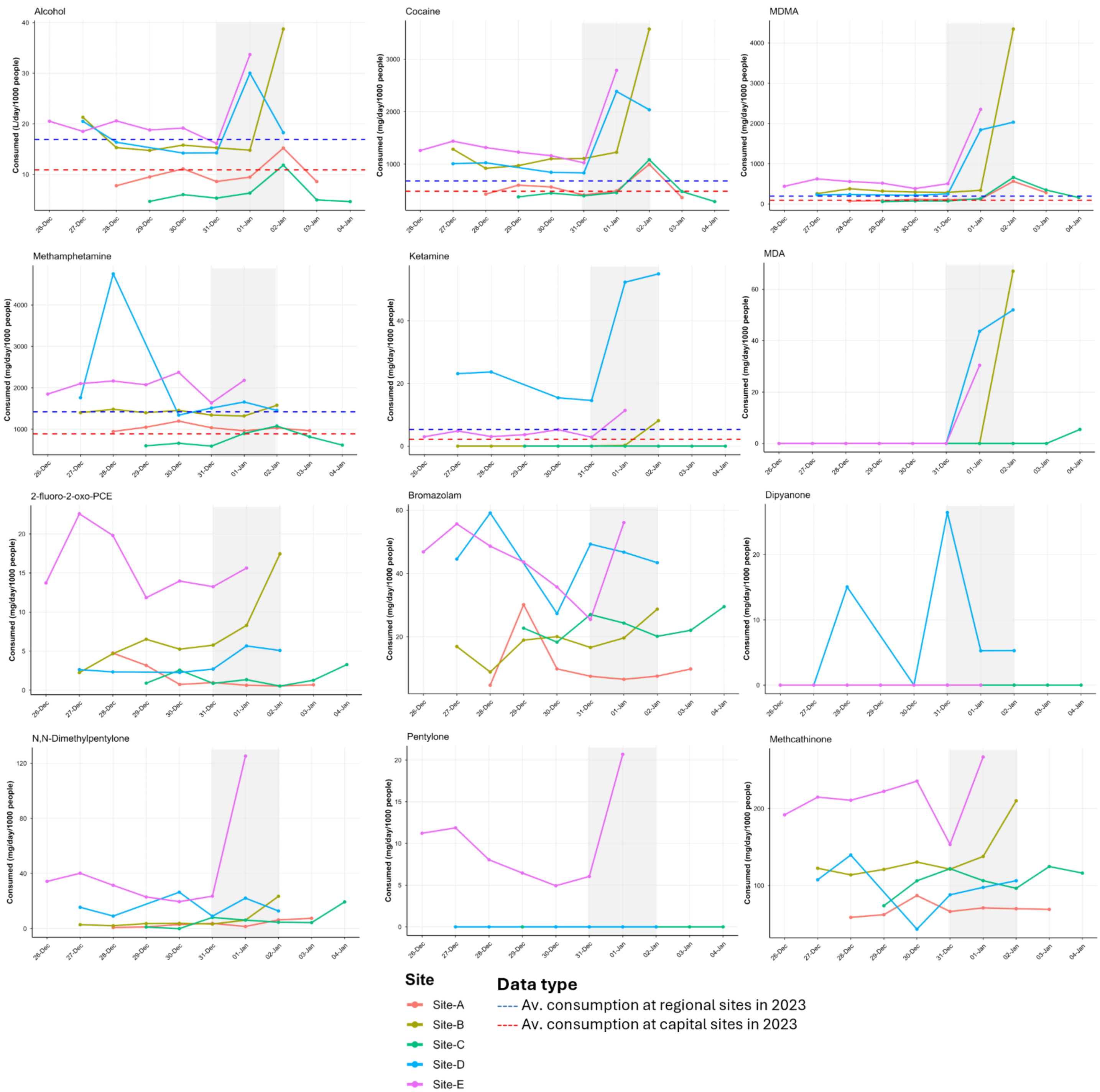


***Figure S7:*** *Trend of consumed drugs during sampling week across five sites by WBE. For consumption of alcohol, it is L/day/1000 people, for illicit drugs & NPS it is mg/day/1000 people. Average consumption at regional (n=2) and capital (n=3) sites in 2023 is based on NDWMP data.*

***Table S6:*** *Summary of drug checking results from the CheQpoint service (April 2024–April 2025). Data are presented as proportions of total psychoactive drug samples analyzed (n=906). Data sourced from CheQpoint 12-month evaluation report* [5].

| SUBSTANCE | DETECTIONS (%) |
|---|---|
| **EXPECTED DRUG TYPES** | |
| **MDMA OR MDA** | 34% |
| **COCAINE** | 17% |
| **KETAMINE OR SIMILAR DISSOCIATIVE** | 11% |
| **METHAMPHETAMINE** | 10% |
| **PSYCHEDELIC** | 8% |
| **BENZODIAZEPINES** | 6% |
| **HEROIN** | 3% |
| **OTHER STIMULANTS** | 2% |
| **OTHER OPIOIDS** | 1% |
| **GHB, GBL & 1,4-B** | 1% |
| **ANALYTICAL OUTCOMES** | |
| **EXPECTED DRUG DETECTED** | 83% |
| **EXPECTED DRUG ONLY (NO ADULTERANTS)** | 70% |
| **UNEXPECTED PSYCHOACTIVE SUBSTANCES** | 10% |
| **INACTIVE FILLERS PRESENT** | 14% |
| **NO DRUG DETECTED** | 3% |
| **NPS IN UNEXPECTED DRUGS** | |
| **BENZOCAINE** | Detected |
| **BROMAZOLAM** | Detected |
| **DIMETHYLPENTYLONE** | Detected |
| **ETHYLBROMAZOLAM** | Detected |
| **ETIZOLAM** | Detected |
| **METHYLPHENIDATE** | Detected |
| **NITAZENES** | Detected |
| **PHENACETIN** | Detected |
| **PROTONITAZENE** | Detected |
| **TILETAMINE** | Detected |
| **2C-B** | Detected |
| **2′-FLUORO-2-OXO-PCE** | Detected |
| **3-MMC** | Detected |
| **4-CMC** | Detected |

## References

1. Gao J, Li J, Jiang G, Yuan Z, Eaglesham G, Covaci A, Mueller JF, Thai PK. Stability of alcohol and tobacco consumption biomarkers in a real rising main sewer. Water research. 2018;138:19–26.

2. Chen S, Bade R, Tscharke B, Hall W, Thai P, He C, Ahmed F, Zheng Q, Choi PM, O'Brien JW, Mueller JF. Assessing daily patterns in stimulant use during the COVID-19 pandemic in Melbourne, Australia using wastewater analysis. Journal of Hazardous Materials. 2024 Sep 5;476:135130. doi:10.1016/j.jhazmat.2024.135130

3. Laimou-Geraniou M, Nadarajan D, Bade R, Heath E. Spatiotemporal trends of new psychoactive substances in Slovenia through influent wastewater analysis. Journal of Hazardous Materials Advances. 2025 Aug 1;19:100781. doi:10.1016/j.hazadv.2025.100781

4. Australian Criminal Intelligence Commission. National Wastewater Drug Monitoring Program : Report 23 [Internet]. 2024 [cited 2025 Mar 23]. Report no.: 23. Available from: www.acic.gov.au

5. CheQpoint. 12 Month Evaluation Report [Internet]. Queensland Health, QuIHN, QuIVAA, and The Loop Australia; 2025 [cited 2026 Apr 14]. p. 1–12. Report VI.I. Available from: https://www.theloop.org.au/reports
